# Effect of *Clostridium perfringens* type D toxin on *Caenorhabditis elegans*

**DOI:** 10.1016/j.namjnl.2025.100038

**Published:** 2025-07-29

**Authors:** Ana María Delia Pereyra, Ximena Blanco Crivelli, Mariana Sanin, Adriana Bentancor, Cecilia Cundon

**Affiliations:** Universidad de Buenos Aires, Facultad de Ciencias Veterinarias, Instituto de Investigaciones en, Epidemiología Veterinaria. Microbiología. Chorroarín 280, Ciudad Autónoma de Buenos Aires, CP1427, Argentina

**Keywords:** *Clostridium perfringens* type D, *Caenorhabditis elegans*, CPD toxin, In vivo model

## Abstract

The search for an alternative in vivo diagnostic method for *Clostridium perfringens* toxinotype D (CPD) aimed to evaluate the susceptibility of the nematode *Caenorhabditis elegans* CF512 CPD as a substitute for conventional biological models. The LD50 of the activated filtrate from CPD strain 426 was determined using a 96-well plate method over 23 h. We performed seroneutralization test with polyclonal anti-CPD toxin to determine the ND50. We observed significant differences between the toxin as the only factor and the toxin combined with polyclonal antibodies between 11 and 14 h after the test began. The evaluation of CPD toxin toxicity, as indicated by the survival or death of the nematode, suggests the potential utility of this model for future diagnostic applications. Although these initial findings provide insight into the CPD toxin's effect in this model, other toxins in the filtrate caused delayed effects. Future investigations employing purified toxins are expected to yield more precise data regarding mortality and neutralization. This study constitutes the inaugural evaluation of CPD effects within the *C. elegans model*, thereby offering direct implications for the biological diagnosis of this toxin and potentially enhancing the accuracy and efficacy of enterotoxemia detection and treatment.

## 1 Introduction

The genus *Clostridium* consists of straight, Gram-positive, anaerobic bacilli extensively distributed in soil and the digestive tracts of animals and humans. *Clostridium perfringens* produces a variety of toxins and enzymes, encoded by over twenty-three virulence genes (Kiu and Hall, 2018). *C. perfringens* strains are classified into seven toxinotypes (A–G) based on the presence or absence of various proteinaceous toxins such as alpha, beta, epsilon, iota, enterotoxin CPE, and necrotic toxin netB. (Table 1). Strains secrete these toxins into the extracellular environment during the exponential growth phase. Additionally, this classification includes the production of enterotoxin and a necrotic toxin like toxin B (Rood et al., 2018).

**Table 1:** *C. perfringens* toxinotyping scheme.

Toxinotype	CPA	CPB	ETX	ITX	CPE	NetB
A	+	–	–	–	–	–
B	+	+	+	–	–	–
C	+	+	–	–	+/-	–
D	+	–	+	–	+/-	–
E	+	–	–	+	+/-	–
F	+	–	–	–	+	–
G	+	–	–	–	–	+

Modified from Pawaiya y col., 2020.

*C. perfringens* type D toxin (CPD toxin) is synthesised by *C. perfringens* types B and D and is encoded on plasmids associated or not with additional virulence genes (Popoff, 2011; Navarro et al., 2018). CPD toxin is produced as an inactive protoxin with an approximate molecular weight of 33 kDa. To acquire its active form (27 kDa weight), the protoxin is activated by enzymes such as trypsin, chymotrypsin and the λ- protease produced by *C. perfringens* (Cavalcanti et al., 2004; Cole et al., 2004). CPD toxin-producing type D strains of *C. perfringens* are responsible for enterotoxemia in sheep, goats, and cattle, and also cause necrotic enteritis in a range of animals, including ruminants and poultry. However, they are not associated with spontaneous disease in humans (Lebrun et al., 2010; Uzal et al., 2018). Among the toxins produced by species of the genus *Clostridium*, CPD toxin is the third most potent, following botulinum and tetanus toxins. The lethal dose 50 (LD50) of CPD toxin is 100 ng/kg when administered intraperitoneally to mice. Researchers have proposed four candidate proteins as CPD toxin receptors: caveolin-1 and caveolin-2 (CAV1 and CAV2), the cellular receptor for hepatitis A virus (HAVCR1), and myelin and lymphocyte protein (MAL) (Ivie et al., 2011; Fennessey et al., 2012; Rumah et al., 2015). The definitive diagnosis of enterotoxemia involves the detection of CPD toxin in intestinal fluid, tissue, culture supernatants, and other body fluids. Although techniques such as immunohistochemistry and mass spectrometry can identify the toxin, they cannot determine the biological activity of CPD toxin (Alam et al., 2012; Bos et al., 2005; Dupré et al., 2015; Miclard et al., 2009; Seyer et al., 2012). Seroneutralization test in mice is a standard method used to detect CPD toxin in biological samples and to assess the potency of clostridial vaccines.

Methods that use animals as biological reagents have driven ongoing research into alternative approaches. *Caenorhabditis elegans* is a cosmopolitan nematode that lives in decaying organic matter, whose similarities to the cellular and molecular processes of animals make it a versatile model. *C. elegans* expresses proteins CAV-1 and CAV-2, which could internalize bacterial toxins such as CPD toxin (Sato et al., 2006). The aim of this study was to determine the susceptibility of *C. elegans* to CPD toxin in both its protoxin and active forms, thereby establishing the utility of the animal model as a biological detection reagent.

## 2 Material and methods

### 2.1 Strains and culture conditions

Three CPD toxin-producing *C. perfringens* strains 426, SG (*C. perfringens* type D), and B2 (*C. perfringens* type B) from the Microbiology Culture Collection of FCV-UBA were used in this study. The strains were cultured on brain heart infusion agar (Britania Laboratories) supplemented with 5 % horse blood under anaerobic conditions (Oxoid jar with Anaero Pack-Anaero® Mitsubishi Gas Chemical Co., Inc) at 37 °C for 48 h. Their identity and purity were confirmed using classical bacteriological techniques (Smith and Holdeman, 1968). The bacterial toxinotype was verified by PCR screening for toxin-associated genes (Meer and Songer, 1997), using *C. perfringens* strains SG and B2 as positive controls. Reactions were carried out using 10 ng of sample DNA template and toxin-specific gene primers *cpd* 3 (5′-CGGTGATATCCATCTATTC-3′) *cpd* 4 (5′-CCACTTACTTGTCCTACTAAC-3´) and *cpb* 1 (5′-TACTATAACTAGAAATAAGACATCAGATGGC-3´) *cpb* 2 (5′-CCACGAGTAGTTTCTGTAAATTTTGTATCCC-3′) along with GoTaq DNA Polymerase (Promega, Madison, WI, USA). PCR cycle parameters were as follows: initial denaturation at 94 °C for 5 min, followed by 28 cycles each of 93 °C for 60 s, 48 °C for 60 s, and 72 °C for 2 min, with a final extension period at 72 °C. Upon completion of PCR, 10 µl of product was run on a 1.2 % agarose gel containing ethidium bromide (Promega, Madison, WI, USA). For subsequent assays, the *C. perfringens* strain 426 was used. The production of protoxin was confirmed in TGY (tryptone 10 g/L, glucose 1 g/L, yeast extract 5 g/L, NaCl 5 g/L, and cysteine hydrochloride 0.5 g/L) under anaerobic conditions, incubating at 37 °C for 6 h by SDS-PAGE, including TGY broth as a negative control and Blue Plus® Protein Marker (14–100 kDa) (TransGen Biotechnology, Beijing, China).

### 2.2 Toxicity assessment

The assays were conducted in NGM medium without OP50. Sensitivity was analyzed based on survival, escape response, and motility alterations. Live and dead specimens were evaluated using a LEICA® EZ4 10x stereomicroscope. The survival of *C. elegans* was monitored every hour for 28 h based on their reaction to touch stimulus using a platinum loop. The avoidance behaviour was noted as the nematodes moved away from the toxin inoculation site, including cases where they left the plate entirely. Four different methods were evaluated in triplicate.

Method 1 - Discs: Sterile 6 mm diameter Whatman No 5 filter paper discs were inoculated with 20 µl of the respective filtrates (either toxins or protoxins). Each was placed onto a 5 cm NGM plate. Subsequently, 10 nematodes were transferred onto each plate, and survival and/or avoidance responses in relation to the disc were evaluated.
Method 2 - Perforations: Perforations were made in the NGM agar using an agar punch. Each well was filled with 100 µl of the filtrate, and 10 nematodes were then transferred into each well. Survival and/or avoidance responses relative to the well were subsequently monitored.
Method 3 - Dispersion: 200 µl of the filtrate were spread evenly onto 5 cm NGM plates. Subsequently, 10 nematodes were transferred onto each plate, and survival and/or avoidance effects were determined.
Method 4 - 96-well Microplate Assay: A 96-well microplate with U-shaped wells, without NGM, was used. Each well was inoculated with 200 µl of the respective filtrate in triplicate. Subsequently, 10 nematodes were transferred into each well and survival and/or avoidance responses were evaluated.

### 2.3 Seroneutralization

Polyclonal anti-CPD toxin antibodies of *C. perfringens* (NR-865) (1 µg/µl), provided by BEI Resources, NIAID, NIH, were used as described by Robertson and McClane (2011). The antibodies were serially diluted in PBS using a 10-fold series, following NIAID and NIH guidelines for in vitro assays, such as Western Blot and ELISA. Antibody dilutions ranging from 10⁻⁴ to 10⁻⁶ were combined with the toxin in vitro to neutralize the *C. perfringens* toxin. Subsequently, 10 CF512 L4 larvae were exposed to each toxin-antibody dilution for 23 h. The model was later refined using 2-fold dilutions to compare the effects of the toxin alone with those of the neutralized toxin.

### 2.4 Determination of LD50 and ND50

The assay duration was established, and successive 10-fold dilutions of the activated filtrate containing the toxin were prepared. The LD50 value was determined at 23 h using the Reed and Muench method. Based on this result, the ND50 was assessed using concentrations of 1 LD50, 10 LD50, and 100 LD50, with appropriate controls including the toxin alone and the antibody.

### 2.5 Results analysis

The survival of *C. elegans* exposed to protoxin, protoxin with penicillin, toxin, TGY, and PBS over time (in hours), along with the SN parameter, was analyzed using the proportion difference method.

## 3 Discussion and conclusions

Enterotoxemia in ruminants caused by *C. perfringens* types B and D is due to the production of a combination of minor and major toxins, with CPD toxin being the most prominent. This disease leads to significant economic losses. The traditional method for diagnosing enterotoxemia has been based on the isolation and identification of the causative pathogen (Smith and Holdeman, 1968). More modern approaches, such as the VITEK 2 system followed by RT-PCR for genotyping, can also be employed (Alsaab et al., 2021). However, the mere isolation and identification of *C. perfringens* alone is insufficient. Genotyping can detect the toxinotype, but it does not confirm toxin production or potency (Uzal & Songer, 2008). There are detection methods for the toxin, including commercial ELISA kits (MULTISCREEN ELISA, Bio-X Diagnostics S.A.), with a detection limit of 10 ng/ml (Jones et al., 2015). Due to the high sensitivity of ELISA, it has been suggested that it may detect low toxin concentrations, even those insufficient to cause disease (Uzal et al., 2003). To reflect the in vivo toxicological potential, MDCK cells have been used (Petit et al., 1997). Additionally, *Mus musculus* has been widely used as an experimental model for detecting clostridial toxins (Uzal et al., 2015). The routes of clostridial toxin inoculation employed have been oral (Goldstein et al., 2009; Poorhassan et al., 2021) or intragastric (Goldstein et al., 2009). In the present study, the *C. elegans* model was used, which proved to be sensitive to CPD toxin. The use of nematodes, which are easy to handle and low-cost, positions them as an alternative biological model (Layana et al., 2006). Unlike cell cultures, *C. elegans* possess organised systems, including neural, motor, reproductive, and digestive systems, endocrine signalling, and sensory and motor responses to various stimuli. Additionally, they can be maintained under aerobic conditions without the need for a carbon dioxide chamber, producing a large number of progeny within 2 to 3 days (Hunt et al., 2017). The high conservation of genes and signalling pathways between *C. elegans* and mammals suggests that this non-mammalian animal model is a good predictor of toxicity, provided that Good *C. elegans* Culture Practice (GCeCP) is followed (Hunt et al., 2017). Despite these advantages, it is important to acknowledge the limitations of *C. elegans* compared to mammalian models. Notably, *C. elegans* lacks an adaptive immune system, limiting its ability to fully replicate the host immune response to *C. perfringens* toxins. Additionally, significant physiological differences exist between nematodes and mammals, which may affect the translation of toxicity and pathogenicity results. Therefore, while *C. elegans* provides valuable preliminary insights and serves as a cost-effective screening tool, findings should be interpreted with caution and validated in mammalian models to ensure biological relevance (Markaki and Tavernarakis, 2020; Sun et al., 2020; Yu et al., 2024).

Like other authors, we worked with culture filtrates of *C. perfringens* (Sayeed et al., 2005; Fernandez-Miyakawa et al., 2007). To establish a testing protocol, various methodologies were analyzed, considering the selection of the *C. elegans* strain, the nematode developmental stage, the assay methodology and conditions, the effect based on exposure time, and the analysis method. Similar to Ahamefule et al. (2021), we selected the CF512 mutant, whose fertility is temperature-dependent, allowing for proper synchronization of the larval stage (Ahamefule, C. S. et al., 2021). Regarding the assay conditions, Kim et al. (2014) used NGM plates treated with toxin. In our case, NGM plates treated with filtrates whether through monodiscs soaked in toxin, agar perforations containing the toxin, or by spreading the filtrate over the agar did not allow for the clear definition of toxin effects due to the nematodes escaping. This effect was not observed in assays using multi-well plates and liquid media, as reported by other authors (JebaMercy et al., 2015). Our assays included 10 L4 individuals per well in 200 µl of solution. Previous studies using liquid media plates differed in the type of plates used (6 or 24 wells), the number of exposed larvae (n: 30), developmental stage (L1 or L4), and tested volume (0.5 ml) (Clavijo et al., 2016; Moya et al., 2022). In our study, assessing the observed effect using >10 L4 individuals proved to be limiting. An increase in the number of nematodes can be achieved through experimental unit repetitions, larger volume plates, or by removing dead larvae at each time point and recording mortality. In this work, the expected effect of CPD toxin was lethality, with nematode survival or death being observed. This contrasts with other studies, such as Kim et al. (2014), who reported locomotion impairments due to botulinum toxin action, or lysis and changes in nematode transparency caused by enzymes present in *C. perfringens* filtrates (Cohen and Troemel, 2015; Kirienko et al., 2014; Hummell et al., 2021). Similar to Moya et al. (2022), who conducted toxicity assays over 24 h, the present study evaluated toxicity up to 28 h. During this period, all nematodes exposed to the toxin died, while all controls survived. Significant results were observed starting from 3 h after exposure began.

The lethal effect was not observed with the protoxins tested but was exclusively induced by protoxin activated via trypsin (Hatheway, 1990; Songer, 1996). Although it was possible that the activation of protoxin could have been initiated at some point by the protease activity of the lambda enzyme secreted by the microorganism itself (Alves et al., 2014), this effect was not detected in our assays.

Analysis of the mortality curve across all experiments revealed two plateaus with a consistent number of viable animals for several hours. The first effect was observed at 3 h, and the second at 11 h. After 23 h, the number of live individuals gradually decreased until all had died. This can be explained by the ability of *C. perfringens* to produce up to 30 potential toxins, including major and minor toxins, as well as proteins with enzymatic activity (Hatheway, 1990; Songer, 1996). The different stages observed in the curve are consistent with the presence of multiple toxic and lethal substances affecting the model. It is important to highlight that the major toxin alpha is present in this filtrate. Although it would be plausible to consider that each initial inflection point in the curve is the result of the effects of major toxins, such as alpha and epsilon, there is no evidence to support this relationship. In contrast, the toxic effect of alpha toxin in filtrates not treated with trypsin would not affect the model, as no lethal effect was observed in this assay. The presence of other *C. perfringens* protoxins in the culture, which can be activated by trypsin, is consistent with the activity of sialidases like NanI, which can be activated by trypsin and chymotrypsin from the host during intestinal infection (Wang, 2020).

The susceptibility of *C. elegans* to CPD toxin may be related to the presence of receptors for the toxin. *C. elegans* has two caveolin genes that encode the proteins CAV-1 and CAV-2 (Sato et al., 2006). In particular, CAV-1 and CAV-2 are implicated in the internalization of CPD toxin (Fennessey et al., 2012). In the late larval stages of the nematode, the expression of CAV-1 is restricted to the neuromuscular system and the germ line. It is noteworthy that the intestine of *C. elegans* comprises a tube of epithelial cells with a dense layer of microvilli on its apical membrane, where CAV-2 is located (Parker et al., 2009). The existing evidence for the presence of an CPD toxin receptor in the intestine of *C. elegans* may partially explain the observed results.

The lethal effect of the toxin was detected from 3 h into the assay. Upon performing the seroneutralization, it was observed that the curve shifts, resulting in an increased survival time that is directly proportional to the concentration of the antibody, with significant differences noted between 11 and 14 h of the assay. This confirmed the effect of CPD toxin on *C. elegans*. Finally, the delayed lethality effect may be attributed to non-neutralised toxins or enzymes that can be activated by trypsin (Hatheway, 1990; Petit et al., 1997; Wang, 2020). Given that the neutralization of the toxin was conducted in vitro prior to exposing the toxin-antitoxin complex to the nematodes, a residual effect on the nematodes from exposure to CPD toxin is ruled out, as its effect is observable at various dilutions (Fig. 2).

The ND50 results varied across five orders of magnitude, requiring an increase in antitoxin concentration from 1.2 × 10⁻⁸ to 6 × 10⁻³ to establish the ND50 between concentrations corresponding to 10 LD50 and 100 LD50. The influence of other toxins present in the supernatant may explain why the results for 10 and 100 LD50 do not follow a logarithmic relationship in the ND50 assays, indicating that these effects could be attributed to additional toxins in the filtrate. Accurate determination of the ND50 will depend on the use of a standard toxin, as the data presented pertain to the mixture of toxins produced by *C. perfringens* type D, including CPD toxin. However, the observed neutralizing effect suggests that the CPD toxin in the culture is neutralized, and its impact can be measured at 11 h post-initiation of the assay. Although this study establishes an initial framework for the model's utility in detecting CPD toxin, further assays are required to determine its correlation with conventional animal models. Previous studies have reported that the addition of penicillin to *C. perfringens* cultures, particularly in L forms, enhanced the release of protoxin due to alterations in the cell wall, although biological activity in animal models was not verified (Bentancor, 2001). In the present study, this background was followed, obtaining the toxin from the CPD strain from cultures supplemented with penicillin, which facilitated cell wall disruption and protoxin release. Similar to toxicological diagnostics in conventional animals, this study analyzed the presence of CPD toxin in cultures of a standard strain activated with trypsin, neutralizing CPD toxin with polyclonal antibodies before inoculating it into the *C. elegans* model. The presence of CAV-2 in the nematode's intestine is consistent with its role as a receptor for CPD toxin, as observed in mammalian cells. New assays using purified toxin would enable the standardisation of these studies and establish their correlation with the murine model (Kaito et al., 2020). In this study, the LD50 for the activated filtrate of *C. perfringens* was established using the *C. elegans* model. Additionally, the ND50 for 10 LD50 was determined, demonstrating that CPD toxin induces lethality in CF512. In addition to the lethality caused by CPD toxin, there is a complex of proteins with enzymatic/toxic activity produced by *C. perfringens*. Variations in ND50 when employing 10 or 100 LD50 are influenced by toxic effects in the model, independent of CPD toxin. It is noteworthy that there are no previous studies of the model with the major toxins of *C. perfringens*, making this the first approach to its investigation. In conclusion, this study provides preliminary results that support the utility of *C. elegans* as a reagent for determining the presence of CPD toxin from *C. perfringens* and for assessing its specificity through seroneutralisation. The findings clearly indicate that other toxins not neutralized by the epsilon antitoxin would affect the nematode at a later stage. This represents the first advance in the model's ability to respond to the effects of *C. perfringens* toxins, particularly CPD toxin. Its correlation with conventional experimental animals will allow for the establishment of the robustness of this approach. Given the low complexity of the tested model, its incorporation into toxicological diagnostics is feasible. The utility of *C. elegans* in biomedical research is increasingly recognized, as demonstrated by recent transcriptomic and functional studies (Lee et al., 2024; Kim et al., 2024) and supported by comprehensive reviews on its applications in aging and metabolism (Yoo et al., 2024).

## 4 Results

The identity and purity of *C. perfringens* were confirmed through classical bacteriological methods. The characterization of *C. perfringens* strains producing CPD toxin was verified by detecting the presence of the *cpd* gene using PCR. The *cpb* gene was not detected, except in the positive control.

The production of protoxin and toxin by *C. perfringens* was confirmed through SDS-PAGE analysis. The mutant strains of *C. elegans* were successfully synchronized at the larval stage by temperature control and maintained as sterile L4 larvae at 25 °C.

The results obtained using discs were variable, as some nematodes managed to escape from the plate, while others moved away from the disc, complicating survival assessment.

Regarding the agar perforation method, some nematodes burrowed beneath the agar, hindering estimation of their contact with the filtrate. Additionally, the filtrate volume diffused into the agar over time. When using the spread plate technique with 5 cm NGM plates, further nematode loss occurred due to escape. Finally, using 96-well plates with U-shaped bottoms, the evaluation was successful, as no nematodes were lost due to escape.

### 4.1 Survival of *C. elegans*

**Fig. 1:**
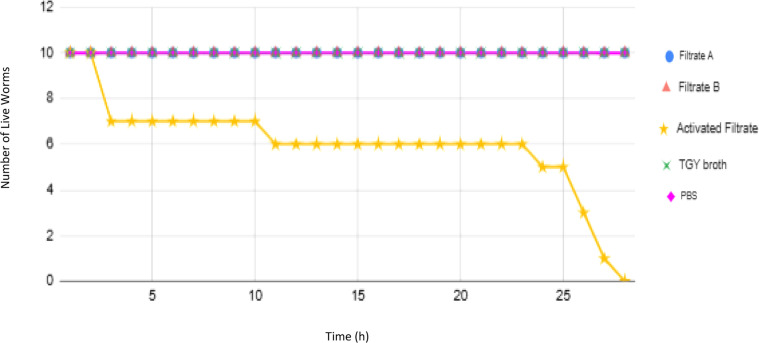
Effect of the protoxins and toxin of *C. perfringens* 426 on the viability of *C. elegans* CF 512 over time.

Filtrates containing protoxin did not cause any nematode mortality throughout the assay period, with the survival rate of nematodes exposed to protoxin and the controls (TGY and PBS) remaining at 100 %. In contrast, a gradual reduction in the number of surviving nematodes was observed in response to the trypsin-activated filtrate starting at 3 h (Fig. 1). Significant differences in survival between nematodes exposed to the toxin and the controls were detected from 11 h onwards. Between 3 and 23 h, two distinct plateaus were observed in the survival curve. From 24 to 28 h, the number of live individuals gradually declined until complete mortality was reached (Table S1).

### 4.2 Determination of LD50

The 1/304 dilution of CPD toxin (activated filtrate) was determined to correspond to the LD50 in *C. elegans* (Table S2).

### 4.3 Seroneutralization

**Fig. 2:**
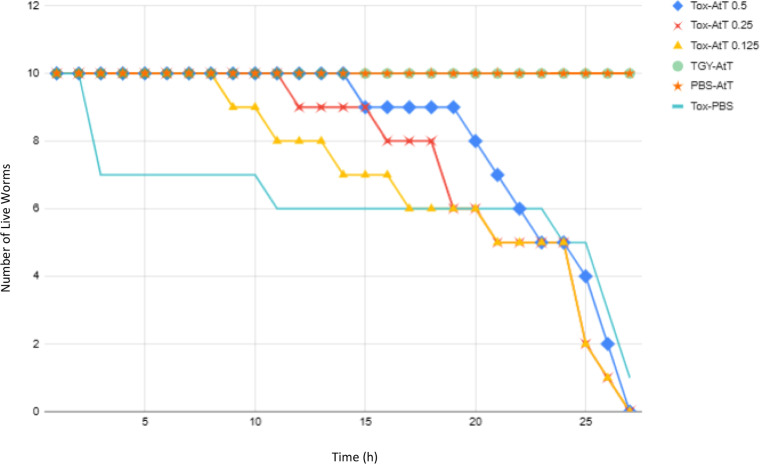
Effect of the protoxins and toxin with antibody of *C. perfringens* 426 on the viability of *C. elegans* CF 512 over time.

The survival rates of 10 L4 nematodes per well are presented in Table S3. The antibody at the tested concentrations (ATC+PBS) did not affect the nematodes. However, the dilution of the antibody used to neutralize the toxin had a noticeable impact on nematode survival (Fig. 2). The survival curve was dependent on antibody concentration, although all nematodes ultimately died by the end of the assay.

The results confirmed the presence and neutralization of CPD toxin. However, the lack of sustained neutralization throughout the entire assay suggests the presence of additional toxins in the filtrate.

### 4.4 Determination of the neutralizing dose

With 1 LD50, 100 % of nematodes survived at antibody dilutions up to 10^−5^, and 90 % survival was observed even at dilutions as high as 10–10. For 10 LD50, the ND50 was 1.2 × 10^−8^, while 100 LD50, the ND50 was 6 × 10^−3^. The results obtained using the Reed and Muench for 1 method are detailed in Table S4.

Table 2

**Table 2:** Seroneutralization according to LD50.

		Live worms		
		1LD50	10LD50	100LD50
**Antitoxin dilutions**	10^−1^	10	9	7
	10^−2^	10	8	5
	10^−3^	10	8	5
	10^−4^	10	7	4
	10^−5^	10	7	4
	10^−6^	9	7	4
	10^−7^	9	7	3
	10^−8^	9	7	2
	10^−9^	9	7	0
	10^−10^	9	7	0
PBS	–	5	0	0

### 4.5 Proportional difference analysis of toxicity assays

At 11 h into the assay, significant differences were observed in the survival of nematodes treated with protoxin compared to the controls, in response to the activated filtrate containing toxin (10 vs 6), with a p-value of 0.043. This effect was significantly neutralized using polyclonal antibodies at a dilution of 0.5 × 10^−4^, showing a significant p-value of 0.043 between 11 and 14 h of the assay. At a dilution of 0.25×10^−4^, a significant p-value of 0.043 was recorded al 11 h. Finally, the 0.125×10^−4^ dilution did not show a significant p-value at any of the time points studied. These results confirm that the antitoxin neutralizes CPD toxin present in the activated *C. perfringens* supernatant, which has a lethal effect on *C. elegans*. Moreover, the delayed onset of the toxic effect suggests either partial neutralization of the toxin or the presence of additional toxins in the filtrate.

## Source of funding

This work was partially supported by PICT BCIE Aplicados II 2021-00139 from ANPCYT.

## CRediT authorship contribution statement

**Ana María Delia Pereyra:** Methodology, Investigation. **Ximena Blanco Crivelli:** Visualization. **Mariana Sanin:** Supervision. **Adriana Bentancor:** Writing – review & editing, Project administration. **Cecilia Cundon:** Conceptualization, Data curation, Formal analysis.

## Declaration of competing interest

The authors declare the following financial interests/personal relationships which may be considered as potential competing interests:

Cecilia Cundon reports financial support was provided by University of Buenos Aires. Cecilia Cundon reports a relationship with University of Buenos Aires Faculty of Veterinary Sciences that includes: employment. Cecilia Cundon has patent pending to Assignee. The authors declare no other relationships or activities that could be perceived as potential conflicts of interest. If there are other authors, they declare that they have no known competing financial interests or personal relationships that could have appeared to influence the work reported in this paper.

## Data Availability

No data was used for the research described in the article.

## Appendix Supplementary materials

Image, application 1
