# Supplementary material for: Effect of Clostridium perfringens type D toxin on Caenorhabditis elegans
Source: NAM J. 2025 Jul 29;1:100038. doi: 10.1016/j.namjnl.2025.100038 (PMC13288649; doi:10.1016/j.namjnl.2025.100038)
Supplement: Supplementary file 1 [file mmc1.docx]

**Table S1. Toxicity of Culture Filtrates of *C. perfringens* D Based on the Number of Live Nematodes**

| **Time (h)** | **Filtrate A** | **Filtrate B** | **Activated Filtrate** | **TGY broth** | **PBS** |
| --- | --- | --- | --- | --- | --- |
| 1 | 10 | 10 | 10 | 10 | 10 |
| 2 | 10 | 10 | 10 | 10 | 10 |
| 3 | 10 | 10 | 7 | 10 | 10 |
| 4 | 10 | 10 | 7 | 10 | 10 |
| 5 | 10 | 10 | 7 | 10 | 10 |
| 6 | 10 | 10 | 7 | 10 | 10 |
| 7 | 10 | 10 | 7 | 10 | 10 |
| 8 | 10 | 10 | 7 | 10 | 10 |
| 9 | 10 | 10 | 7 | 10 | 10 |
| 10 | 10 | 10 | 7 | 10 | 10 |
| 11 | 10 | 10 | 6 | 10 | 10 |
| 12 | 10 | 10 | 6 | 10 | 10 |
| 13 | 10 | 10 | 6 | 10 | 10 |
| 14 | 10 | 10 | 6 | 10 | 10 |
| 15 | 10 | 10 | 6 | 10 | 10 |
| 16 | 10 | 10 | 6 | 10 | 10 |
| 17 | 10 | 10 | 6 | 10 | 10 |
| 18 | 10 | 10 | 6 | 10 | 10 |
| 19 | 10 | 10 | 6 | 10 | 10 |
| 20 | 10 | 10 | 6 | 10 | 10 |
| 21 | 10 | 10 | 6 | 10 | 10 |
| 22 | 10 | 10 | 6 | 10 | 10 |
| 23 | 10 | 10 | 6 | 10 | 10 |
| 24 | 10 | 10 | 5 | 10 | 10 |
| 25 | 10 | 10 | 5 | 10 | 10 |
| 26 | 10 | 10 | 3 | 10 | 10 |
| 27 | 10 | 10 | 1 | 10 | 10 |
| 28 | 10 | 10 | 0 | 10 | 10 |

References: Number of worm/well: 10

Filtrate A: Culture of *C. perfringens* in TGY

Filtrate B: Culture of *C. perfringens* in TGY +PEN

Activated Filtrate: Culture of C. perfringens in TGY + PEN, followed by Trypsin Treatment

##### **Table S2. Determination of the Lethal Dose 50 (LD50) of Activated Filtrate from *C. Perfringens* D strain 426 In *C. elegans***

| **Log Dilution of the Toxin** | **Dead** | **Alive** | **Cumulative Dead** | **Cumulative Alive** | **Total Cumulative** | **% Dead** |
| --- | --- | --- | --- | --- | --- | --- |
| 1 | 9 | 1 | 20 | 1 | 21 | 95,24 |
| 2 | 7 | 3 | 11 | 4 | 15 | 73,33 |
| 3 | 1 | 9 | 4 | 12 | 16 | 25,00 |
| 4 | 1 | 9 | 3 | 21 | 24 | 12,50 |
| 5 | 1 | 9 | 2 | 30 | 32 | 6,25 |
| 6 | 1 | 9 | 1 | 39 | 40 | 2,50 |
| 7 | 0 | 10 | 0 | 49 | 49 | - |

LD50= 304

**Table S3. Seroneutralization of Activated *C. perfringens* D Filtrate with 1/10 Antitoxin Dilutions**

| **Time** | **Activated Filtrate +Antitoxin dilutions** | | | **TGY+ Antitoxin (10^-4^)** | **PBS+ Antitoxin (10^-4^)** |
| --- | --- | --- | --- | --- | --- |
|  | **10^-4^** | **10^-5^** | **10^-6^** |  |  |
| 1 | 10 | 10 | 10 | 10 | 10 |
| 2 | 10 | 10 | 10 | 10 | 10 |
| 3 | 10 | 10 | 9 | 10 | 10 |
| 4 | 9 | 9 | 8 | 10 | 10 |
| 5 | 9 | 8 | 8 | 10 | 10 |
| 6 | 9 | 8 | 8 | 10 | 10 |
| 7 | 8 | 7 | 7 | 10 | 10 |
| 8 | 8 | 7 | 7 | 10 | 10 |
| 9 | 8 | 7 | 7 | 10 | 10 |
| 10 | 7 | 6 | 7 | 10 | 10 |
| 11 | 7 | 6 | 6 | 10 | 10 |
| 12 | 7 | 6 | 6 | 10 | 10 |
| 13 | 7 | 5 | 5 | 10 | 10 |
| 14 | 6 | 5 | 5 | 10 | 10 |
| 15 | 6 | 5 | 4 | 10 | 10 |
| 16 | 6 | 5 | 4 | 10 | 10 |
| 17 | 6 | 4 | 3 | 10 | 10 |
| 18 | 5 | 4 | 3 | 10 | 10 |
| 19 | 5 | 3 | 2 | 10 | 10 |
| 20 | 4 | 3 | 2 | 10 | 10 |
| 21 | 4 | 2 | 1 | 10 | 10 |
| 22 | 3 | 2 | 1 | 10 | 10 |
| 23 | 3 | 1 | 0 | 10 | 10 |
| 24 | 2 | 0 | 0 | 10 | 10 |
| 25 | 2 | 0 | 0 | 10 | 10 |
| 26 | 1 | 0 | 0 | 10 | 10 |
| 27 | 1 | 0 | 0 | 10 | 10 |
| 28 | 0 | 0 | 0 | 10 | 10 |

**Table S4 Seroneutralization of Activated Filtrate of *C. perfringens* D with Dilutions of Antitoxin**

| Time (h) | Tox-AtT 0.5x10^-4^ | Tox-AtT 0.25x10^-4^ | Tox-AtT 0.125x10^-4^ | TGY-AtT | PBS-AtT | Tox-PBS |
| --- | --- | --- | --- | --- | --- | --- |
| 1 | 10 | 10 | 10 | 10 | 10 | 10 |
| 2 | 10 | 10 | 10 | 10 | 10 | 10 |
| 3 | 10 | 10 | 10 | 10 | 10 | 7 |
| 4 | 10 | 10 | 10 | 10 | 10 | 7 |
| 5 | 10 | 10 | 10 | 10 | 10 | 7 |
| 6 | 10 | 10 | 10 | 10 | 10 | 7 |
| 7 | 10 | 10 | 10 | 10 | 10 | 7 |
| 8 | 10 | 10 | 10 | 10 | 10 | 7 |
| 9 | 10 | 10 | 9 | 10 | 10 | 7 |
| 10 | 10 | 10 | 9 | 10 | 10 | 7 |
| 11 | 10 | 10 | 8 | 10 | 10 | 6 |
| 12 | 10 | 9 | 8 | 10 | 10 | 6 |
| 13 | 10 | 9 | 8 | 10 | 10 | 6 |
| 14 | 10 | 9 | 7 | 10 | 10 | 6 |
| 15 | 9 | 9 | 7 | 10 | 10 | 6 |
| 16 | 9 | 8 | 7 | 10 | 10 | 6 |
| 17 | 9 | 8 | 6 | 10 | 10 | 6 |
| 18 | 9 | 8 | 6 | 10 | 10 | 6 |
| 19 | 9 | 6 | 6 | 10 | 10 | 6 |
| 20 | 8 | 6 | 6 | 10 | 10 | 6 |
| 21 | 7 | 5 | 5 | 10 | 10 | 6 |
| 22 | 6 | 5 | 5 | 10 | 10 | 6 |
| 23 | 5 | 5 | 5 | 10 | 10 | 6 |
| 24 | 5 | 5 | 5 | 10 | 10 | 5 |
| 25 | 4 | 2 | 2 | 10 | 10 | 5 |
| 26 | 2 | 1 | 1 | 10 | 10 | 3 |
| 27 | 0 | 0 | 0 | 10 | 10 | 1 |

References: Tox: *C. perfringens* D Filtrate Activated with Trypsin; AtT: antiepsilon toxin; Negative controls: TGY (broth culture); PBS; Positive Control: Activated Filtrate Without Antitoxin

**Table S5.** Neutralizing Dose 50 (ND50) with 1, 10, and 100 LD50

| **LD50** | **Log Dilution antitoxin** | **alive worms** | **dead worms** | **Cumulative Alive** | **Cumulative Dead** | **Total Cumulative** | **%**  **alive** |
| --- | --- | --- | --- | --- | --- | --- | --- |
| 1LD50 | 1 | 10 | 0 | 10 | 5 | 15 | 66,67 |
|  | 2 | 10 | 0 | 20 | 5 | 25 | 80,00 |
|  | 3 | 10 | 0 | 30 | 5 | 35 | 85,71 |
|  | 4 | 10 | 0 | 40 | 5 | 45 | 88,89 |
|  | 5 | 10 | 0 | 50 | 5 | 55 | 90,91 |
|  | 6 | 9 | 1 | 59 | 5 | 64 | 92,19 |
|  | 7 | 9 | 1 | 68 | 4 | 72 | 94,44 |
|  | 8 | 9 | 1 | 77 | 3 | 80 | 96,25 |
|  | 9 | 9 | 1 | 66 | 2 | 68 | 97,06 |
|  | 10 | 9 | 1 | 55 | 1 | 56 | 98,21 |
| 10LD50 | 1 | 9 | 1 | 74 | 1 | 75 | 98,67 |
|  | 2 | 8 | 2 | 65 | 3 | 68 | 95,59 |
|  | 3 | 8 | 2 | 57 | 5 | 62 | 91,94 |
|  | 4 | 7 | 3 | 49 | 8 | 57 | 85,96 |
|  | 5 | 7 | 3 | 42 | 11 | 53 | 79,25 |
|  | 6 | 7 | 3 | 35 | 14 | 49 | 71,43 |
|  | 7 | 7 | 3 | 28 | 17 | 45 | 62,22 |
|  | 8 | 7 | 3 | 21 | 20 | 41 | 51,22 |
|  | 9 | 7 | 3 | 14 | 23 | 37 | 37,84 |
|  | 10 | 7 | 3 | 7 | 26 | 33 | 21,21 |
| 100LD50 | 1 | 7 | 3 | 34 | 3 | 37 | 91,89 |
|  | 2 | 5 | 5 | 27 | 8 | 35 | 77,14 |
|  | 3 | 5 | 5 | 22 | 13 | 35 | 62,86 |
|  | 4 | 4 | 6 | 17 | 19 | 36 | 47,22 |
|  | 5 | 4 | 6 | 13 | 25 | 38 | 34,21 |
|  | 6 | 4 | 6 | 9 | 31 | 40 | 22,50 |
|  | 7 | 3 | 7 | 5 | 38 | 43 | 11,63 |
|  | 8 | 2 | 8 | 2 | 46 | 48 | 4,17 |
|  | 9 | 0 | 10 | 0 | 56 | 56 | - |
|  | 10 | 0 | 10 | 7 | 66 | 73 | - |
